# 5-Aminolevulinic Acid-Guided Glioma Resection During Awake Craniotomy: Systematic Review of Functional Safety and Surgical Benefit

**DOI:** 10.3390/cancers18203365

**Published:** 2026-10-17

**Authors:** Mohammad Mofatteh, Mohammad Sadegh Mashayekhi, John Sinclair, Keyoumars Ashkan

**Affiliations:** 1 School of Medicine, Dentistry and Biomedical Sciences, Queen’s University Belfast, Belfast BT7 1NN, UK; 2 Department of Neurosurgery, Neuro International Collaboration (NIC), London SE5 9RS, UK; k.ashkan@nhs.net; 3 Division of Neurosurgery, Faculty of Medicine, University of Ottawa, Ottawa, ON K1h 8M5, Canada; mmashayekhi@toh.ca (M.S.M.); jsinclair@toh.ca (J.S.); 4 Department of Neurosurgery, Neuro International Collaboration (NIC), Ottawa, ON K1h 8M5, Canada; 5 School of Biomedical Engineering and Imaging Sciences, Faculty of Life Sciences and Medicine, King’s College London, London SE1 7EH, UK; 6 King’s Health Partners Academic Health Sciences Centre, London SE1 9RT, UK; 7 Department of Basic and Clinical Neuroscience, Institute of Psychiatry, Psychology and Neuroscience, King’s College London, London SE5 9RS, UK; 8 Department of Neurosurgery, King’s College Hospital NHS Foundation Trust, London SE5 9RS, UK

**Keywords:** awake craniotomy, awake brain surgery, brain mapping, patient outcome, 5-aminolevulinic acid, 5-ALA, fluorescence-guided surgery, glioma, neurosurgery

## Abstract

**Background:** Maximal safe resection remains the cornerstone of glioma surgery, particularly in eloquent regions where oncologic gain must be balanced against functional preservation. While 5-aminolevulinic acid (5-ALA) fluorescence-guided surgery improves extent of resection in high-grade gliomas under general anesthesia, its role during awake craniotomy (AC) remains underdeveloped. We aimed to evaluate the oncologic and functional outcomes of 5-ALA-guided tumor resection during AC. **Methods:** PubMed, Scopus, and Web of Science were searched from inception to February 2026 for studies reporting original data on AC using 5-ALA following PRISMA guidelines. **Results:** Across ten studies comprising 108 patients, fluorescence positivity was 79.2% in high-grade gliomas (Grade III-IV) and 24.1% in low-grade gliomas (Grade II), among the 83 patients with both grade and fluorescence data available (grade was not reported for 19 patients in one case series, and 6 patients in one cohort study did not receive 5-ALA and were excluded from grade- and fluorescence-stratified analyses). Gross total resection was achieved in 62.2% of patients with evaluable extent-of-resection data (56.5% of the full cohort), reaching 66.7% in the largest comparative cohort. Transient aphasia occurred in 32.6% of evaluable patients, while permanent aphasia and persistent hemiparesis were observed in 14.6% and 16.9%, respectively. In multiple studies, resection was intentionally halted despite persistent fluorescence when functional mapping identified an eloquent cortex, underscoring that mapping remained the decisive determinant of surgical boundaries. No study reported conversion to general anesthesia. **Conclusions:** 5-ALA-guided resection during AC appears feasible and clinically useful, particularly for high-grade gliomas in eloquent regions. Permanent neurological morbidity was observed in a minority of patients, within a range broadly consistent with awake glioma surgery generally, but the absence of a non-5-ALA comparator group means that functional safety relative to awake mapping alone cannot yet be established with confidence. Across the available literature, 5-ALA consistently enhances intraoperative tumor visualization, supports identification of residual fluorescent disease, and integrates well with cortical and subcortical mapping without clear evidence of increased permanent neurological morbidity. Although functional mapping remains the decisive determinant of the final resection boundary, the available evidence supports 5-ALA as a valuable complementary adjunct in onco-functional glioma surgery.

## Simple Summary

Gliomas are brain tumors that can grow close to areas responsible for important functions such as speech and movement, making complete removal challenging. Awake brain surgery allows surgeons to monitor these functions during tumor removal, while 5-aminolevulinic acid is a substance that causes tumor tissue to glow under a special light and may help surgeons identify tissue that would otherwise be difficult to see. This systematic review examined whether combining these techniques can improve tumor removal while preserving neurological function. Ten studies involving 108 patients were included. Fluorescence was particularly useful with high-grade gliomas and helped surgeons identify residual tumor during surgery. Importantly, functional brain mapping remained the main factor determining when tumor removal should stop, even when fluorescent tissue remained. Overall, the findings suggest that fluorescence-guided surgery can be safely integrated with awake brain surgery, although larger, well-designed studies are needed to determine its additional benefits and identify which patients are most likely to benefit.

## 1. Introduction

Maximal safe resection of the tumor tissue remains the cornerstone of glioma surgery. The extent of resection (EOR) correlates strongly with both progression-free and overall survival in low- and high-grade gliomas [1,2,3,4,5]. Achieving gross total resection (GTR) near eloquent cortical or subcortical areas remains challenging, as aggressive removal risks permanent neurological deficit [6,7,8,9,10]. Awake craniotomy (AC) with direct cortical and subcortical mapping enables maximal tumor removal while preserving motor, language, and cognitive networks [11,12,13,14,15].

**Figure 1:**
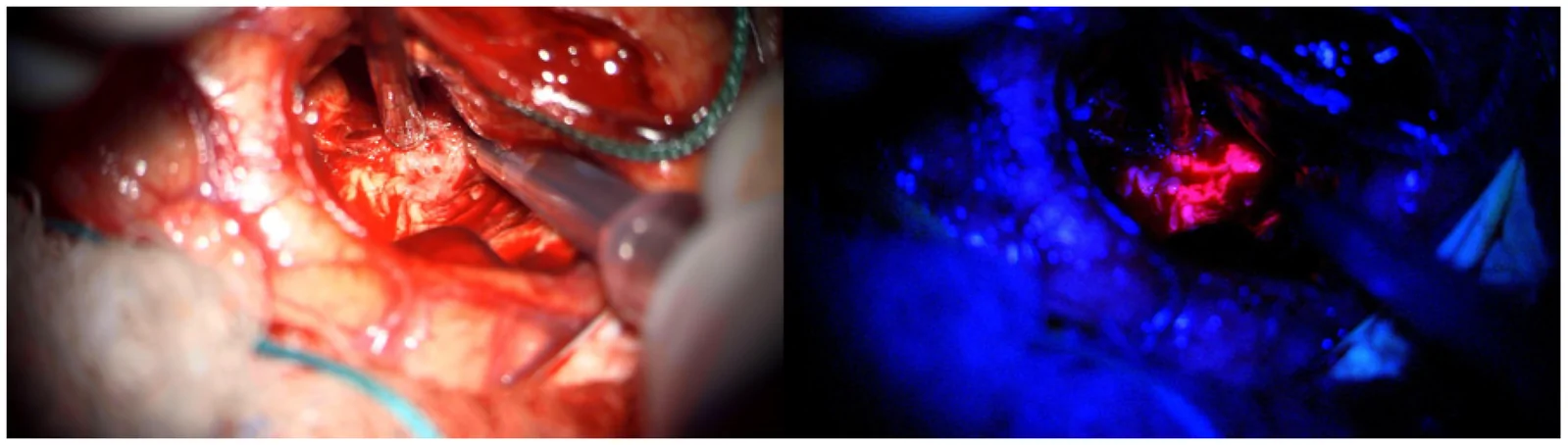
Intraoperative images during resection of a left frontal glioblastoma in a 64-year old female using 5-ALA by one of the authors. (**Left**): White-light view of surgical cavity demonstrating limited distinction between tumor and surrounding healthy brain tissues. (**Right**): Demonstration of the same view under blue-light fluorescence, with residual tumor highlighted in bright pink. ALA, aminolevulinic acid.

Parallel advances in fluorescence-guided surgery using 5-aminolevulinic acid (5-ALA) have improved intraoperative tumor visualization and EOR under general anesthesia [16,17,18]. Following oral administration, 5-ALA is metabolized to protoporphyrin IX, emitting red-pink fluorescence under blue light and delineating malignant tissue [19,20,21,22]. Randomized trials have shown that 5-ALA improves complete resection rates and progression-free survival in glioblastoma, making it a routine adjunct in high-grade glioma management [23,24]. Figure 1 demonstrates application of 5-ALA in resection of a right frontal glioblastoma in a 64-year-old female by one of the authors. Under a standard white-light microscope, the distinction between tumor and surrounding brain tissue is limited; however, tumor is highlighted by pink fluorescence under blue light in the same surgical cavity, facilitating maximum resection of neoplastic tissue.

Awake mapping and 5-ALA fluorescence offer distinct but highly complementary intraoperative information. Whereas direct electrical stimulation defines the functional limits of safe resection, 5-ALA enhances visualization of metabolically active tumor tissue and may improve identification of residual disease, particularly in high-grade gliomas [25,26]. Although fluorescence cannot substitute for mapping in eloquent cortex and may be less sensitive in lower-grade gliomas, its integration into awake surgery offers an appealing onco-functional strategy in which tumor visualization and functional preservation are pursued simultaneously. Conversely, non-fluorescent infiltrative tumor may extend beyond visible margins, particularly in lower-grade gliomas, limiting the reliability of fluorescence as a sole guide [27].

Beyond intraoperative direct electrical stimulation, navigated transcranial magnetic stimulation (nTMS) has become an established preoperative adjunct, enabling non-invasive mapping of motor and language-eloquent cortex before surgery and informing both surgical approach planning and patient counselling regarding anticipated functional risk [28,29]. While nTMS does not replace intraoperative mapping, its integration with fluorescence-guided awake surgery, for example by pre-identifying eloquent cortex to be avoided or specifically tested intraoperatively, represents a further potential point of convergence between preoperative planning technologies and intraoperative onco-functional strategies and is discussed further in Section 4.6.

The literature examining 5-ALA specifically within the context of AC, however, remains limited, consisting predominantly of small retrospective series and case reports. Data regarding fluorescence behavior during cortical versus subcortical phases, concordance with histopathology in eloquent regions, and the influence of 5-ALA on surgical stopping points during awake procedures are sparse.

Other adjuncts, including intraoperative MRI, may further refine resection, but 5-ALA remains attractive because it is of lower cost and more readily available, is immediately actionable at the operative field, can guide selective continuation of resection in non-eloquent fluorescent regions, and may increase surgeon confidence at the tumor–brain interface [30,31,32,33,34]. Accordingly, the key question is not whether mapping or fluorescence should take priority—mapping clearly must—but how much additional practical and oncologic value fluorescence provides when used alongside awake functional monitoring [35,36,37].

Despite growing use of 5-ALA in functional neuro-oncology, no prior systematic review has specifically evaluated its integration within awake mapping paradigms. This systematic review synthesizes available evidence on the combined use of 5-ALA and awake functional mapping in glioma surgery, focusing on fluorescence behavior, oncologic and functional outcomes, and intraoperative decision-making.

## 2. Materials and Methods

### 2.1. Data Source and Search Strategy

We conducted this systematic review following the Preferred Reporting Items for Systematic Reviews and Meta-Analysis (PRISMA) guidelines [38] to identify published literature on AC in patients using 5-ALA. PubMed, Scopus, and Web of Science databases were searched from inception to 21 February 2026 for relevant articles. The following Boolean terms were used for the search: (“awake craniotomy” OR “awake brain surgery” OR “awake neurosurgery” OR “awake brain mapping” OR “awake tumor resection” OR “craniotomy while awake”). Given the rarity of 5-ALA usage in AC, a deliberately broad search strategy centered on AC was used to maximize sensitivity, and no database-specific string included a 5-ALA-related term (e.g., “5-aminolevulinic acid,” “5-ALA,” “Gliolan”). This was a deliberate sensitivity-maximizing decision rather than an omission, because 5-ALA use during AC is infrequently mentioned as a distinct searchable concept. Thus, restricting the search to 5-ALA-specific terms risked excluding eligible reports in which 5-ALA administration was described only in the full text, figures, or Supplementary Materials rather than in the title or abstract. To mitigate residual sensitivity loss from this broad strategy, reference lists of all included studies were additionally hand-screened (Section 2.3) as a backward-citation cross-check. The complete, verbatim search strings used for PubMed, Scopus, and Web of Science are reproduced in full in Supplementary Table S1 and are available on request for independent reproduction.

### 2.2. Inclusion and Exclusion Criteria

Eligible studies met these criteria: (1) original human data; (2) English language; (3) AC performed with 5-ALA administration; (4) sufficient intraoperative and postoperative detail for data extraction. Studies were excluded if they included only general anesthesia cases, combined multiple fluorescence agents, or lacked extractable clinical information. Prospective registration (e.g., with PROSPERO) was considered before the search was initiated. A formal protocol was not registered publicly because the anticipated evidence base was expected to be very small and dominated by single-patient case reports, for which we judged a quantitative, meta-analytic protocol to be premature; however, eligibility criteria, the search strategy, and the outcome domains of interest (extent of resection, fluorescence characteristics, functional outcomes, and intraoperative decision-making) were specified in an internal a priori protocol before title and abstract screening began and were not modified thereafter. No post hoc changes were made to eligibility criteria, outcome domains, or the analytic approach after data extraction commenced; the descriptive (rather than meta-analytic) synthesis strategy described in Section 2.4 was therefore a pre-specified decision rather than a post hoc adaptation to the data obtained. We acknowledge the absence of public registration as a methodological limitation (Section 4.7).

### 2.3. Article Selection Process

Initial screening of titles and abstracts was independently performed by two reviewers (M.M. and M.S.M.). Potentially eligible articles underwent full-text review for final inclusion. Discrepancies were resolved by consensus. Reference lists of all included studies were additionally screened to identify relevant articles not captured by the database search.

### 2.4. Data Extraction and Synthesis

Two reviewers (M.M. and M.S.M.) independently extracted study and patient-level variables, including demographics, tumor features, operative technique, fluorescence findings, and outcomes. The methodological quality of included case reports was assessed using the Joanna Briggs Institute (JBI) Critical Appraisal Checklist for Case Reports. Disagreements were resolved by consensus.

Where sufficiently comparable data were available across studies, categorical outcomes were summarized as counts and proportions using the number of patients with available relevant data as the denominator. Because the evidence base combines single-patient case reports, a retrospective case series, and prospective/retrospective cohorts with substantially different evidentiary weight, all pooled percentages reported in this review represent simple patient-level descriptive aggregation across the included studies and must not be interpreted as weighted summary estimates or as the product of formal evidence synthesis. 

## 3. Results

### 3.1. Study Selection

**Figure 2:**
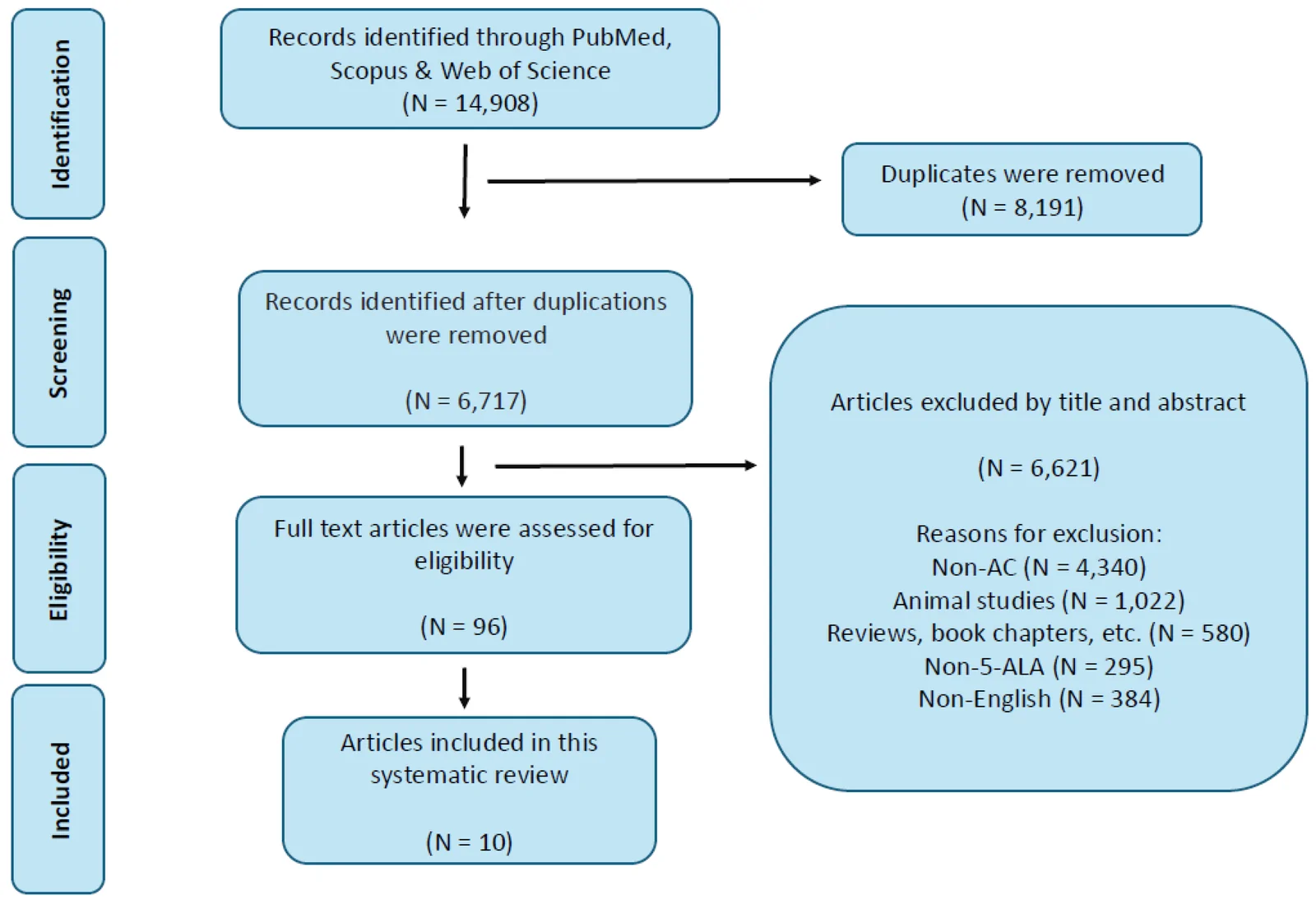
A flowchart of the current study demonstrating the identification, screening, eligibility, and inclusion process according to the Preferred Reporting Items for Systematic Reviews and Meta-Analyses (PRISMA). AC, awake craniotomy; ALA, aminolevulinic acid.

A total of 14,908 records were identified. After deduplication and screening, ten studies comprising 108 patients were included (Figure 2). Publication years ranged from 2015 to 2025 and originated mainly from Europe and North America.

### 3.2. Study Characteristics

A total of ten studies with 108 patients undergoing 5-ALA-guided AC were included [39,40,41,42,43,44,45,46,47,48]. Six studies were single-patient case reports, one was a retrospective case series (n = 19), one was a prospective observational cohort (n = 40), and one was a retrospective comparative cohort (n = 42). Larger cohort studies accounted for 101 of 108 patients (93.5%), whereas case reports contributed 7.4%. Recurrent tumors were reported in 16 of 108 patients (14.8%), most frequently in the retrospective case series and comparative cohort (Table 1).

### 3.3. Quality Assessment of Included Studies

Quality assessment using the Joanna Briggs Institute (JBI) critical appraisal tools [49] was performed at the individual item level for each study, using the 8-item JBI Case Report Checklist, the 10-item JBI Case Series Checklist, and the 11-item JBI Cohort Checklist, as appropriate to the study design. Because different JBI instruments contain different items addressing different methodological domains, item-level responses are scored individually for each study, rather than being collapsed into a single global percentage score, so that the specific domain(s) underlying each study’s methodological limitations can be identified. Table 2a below reproduces the exact item wording used for each checklist, and Table 2b provides a fully worked item-level example for one representative case report (Corns et al. [39]) to illustrate the appraisal approach applied to all ten studies. An overall qualitative risk-of-bias impression (low/moderate/high) is retained for each study to aid rapid interpretation, but this impression is derived from, and should be read together with, the item-level judgements rather than from a numerical percentage threshold.

### 3.4. Patient Demographics and Baseline Characteristics

Across the 108 patients, age ranged from 32 to 73 years, with median or mean ages in cohort studies between 42 and 54 years. In total, 62 patients were male (57.4%), and 46 were female (42.6%). Preoperative Karnofsky Performance Status (KPS) was reported in 49 patients, with a median of 90 and a minimum reported value of 70. Preoperative neurological deficits were common: aphasia in 21 patients (19.4%), seizures in 31 (28.7%), motor deficits in 37 (34.3%), and cognitive impairment in 10 (9.3%). Dominant hemisphere tumors accounted for approximately three-quarters of reported cases. Special populations included one patient with autism spectrum disorder (0.9%), one elderly patient aged ≥70 years (0.9%), and multiple reoperations in 13 patients (12.0%) (Table 3).

### 3.5. Tumor Characteristics

High-grade gliomas predominated among patients with reported grade. Of 83 patients with tumor grade and 5-ALA both reported, 37 (44.6%) were WHO Grade IV, 15 (18.1%) were Grade III, 1 (1.2%) was a high-grade glioma of unspecified subgrade (Matei et al. [46]), and 29 (34.9%) were Grade II; one further patient (Price et al. [45]) had astroblastoma, a non-astrocytic entity not assigned a II–IV grade. Grade was not reported for 19 patients in the Skjøth-Rasmussen case series (tumors described only as “suspected high-grade glioma” without a specific WHO grade), and 6 patients in the Goryaynov cohort did not receive 5-ALA and are excluded from grade- and fluorescence-stratified analyses throughout.

IDH status was variably reported but most Grade IV tumors were IDH-wildtype. Eloquent region involvement was documented in 93 patients (86.1%), most commonly affecting frontal, temporal, insular, and perisylvian regions. Contrast enhancement was present in all Grade IV tumors and in the majority of Grade III tumors. (Table 4).

### 3.6. Protocols Used in Awake Craniotomy 

The sleep-awake-sleep technique was used in 100 of 108 patients (92.6%), while fully awake protocols were used in 8 patients (7.4%). Direct cortical stimulation was used in all cases (100%). Positive cortical or subcortical mapping responses were observed in approximately half of patients in larger cohorts. Intraoperative seizures occurred in four patients (3.7%), and one patient (0.9%) developed a postoperative hematoma requiring reoperation. No study reported conversion to general anesthesia (Table 5).

### 3.7. 5-ALA Fluorescence Characteristics

Standard 5-ALA dosing (20 mg/kg administered 2–3 h preoperatively) was used in the majority of cases. Among the 83 patients with both 5-ALA exposure and tumor grade (or astroblastoma histology) reported, overall fluorescence positivity was observed in 50 (60.2%). Among high-grade gliomas (Grade III–IV, n = 53), fluorescence was present in 42 (79.2%), whereas among low-grade gliomas (Grade II, n = 29), fluorescence was present in 7 (24.1%). Grade and fluorescence status could not be determined for the 19 Skjøth-Rasmussen patients or for the 6 Goryaynov patients who did not receive 5-ALA; these 25 patients are therefore excluded from the denominators above but retained in the overall review sample (n = 108).

Bright fluorescence was almost exclusively observed in Grade IV tumors. Fluorescence–histology concordance was consistently reported where evaluated. In several cases, persistent fluorescence in eloquent regions was overruled by functional mapping to preserve language or motor function (Table 6).

### 3.8. Extent of Resection and Adjuncts

GTR was achieved in 61 of 98 patients with evaluable extent-of-resection data (62.2%; 56.5% of the full cohort of 108). Subtotal resection exceeding 90% was reported in 24 patients (22.2%), while biopsy or partial resection was performed in 6 patients (5.6%). These 6 biopsy cases, all from the Goryaynov cohort [43], reflect a distinct, pre-specified application of 5-ALA: targeted biopsy of focal fluorescent areas within otherwise non-contrast-enhancing, presumed low-grade tumors, performed specifically to identify occult anaplastic transformation rather than to guide maximal resection. This use case is conceptually complementary to, but distinct from, 5-ALA’s role in guiding resection extent elsewhere in this review and is retained because it falls within the review’s eligibility criteria (5-ALA administration during AC), even though its surgical objective differs from resection maximization.

In the largest comparative cohort, GTR was achieved in 66.7% of patients. Achieving a 5-ALA-free cavity was significantly associated with GTR. Adjuncts frequently used alongside 5-ALA included neuronavigation, diffusion tractography, and intraoperative MRI (16.0% of patients). In eloquent regions, functional mapping frequently determined the stop point even when residual fluorescence was visible (Table 7).

### 3.9. Functional Outcomes and Survival

Transient postoperative aphasia occurred in 29 of 89 evaluable patients (32.6%), while permanent aphasia was reported in 13 patients (14.6%). Persistent hemiparesis occurred in 15 patients (16.9%). These denominators exclude the 19 Skjøth-Rasmussen [40] patients, for whom transient versus permanent deficit status was not separately reported (only “no exacerbation of neurological condition” overall); all other studies, including both Goryaynov [43] treatment cohorts and both Tagle-Vega [48] treatment subgroups, are included using the exact patient-level counts reported in each primary source.

In contrast, none of the single-patient case reports described permanent neurological deficits. Postoperative median KPS, where reported, remained 90, with improvement documented in approximately one-fifth of reported cases. Follow-up duration ranged from 3 to 15 months. Recurrence was reported in 14 of 22 patients (63.6%) in studies providing follow-up data. Across studies, authors consistently concluded that 5-ALA enhanced tumor visualization, but functional mapping remained the decisive determinant of resection boundaries in eloquent cortex (Table 8).

To make the contribution of each study design tier transparent, headline outcomes are additionally presented stratified by study design in Table 9. No comparative pooled effect estimates, meta-analytic weighting, or between-design statistical comparisons were calculated.

A structured comparison of stimulation modality, task batteries, and resection-stopping criteria across studies is provided in Table 10.

## 4. Discussion

This systematic review synthesizes the currently available evidence on utility of 5-ALA-guided resection during AC for gliomas in eloquent regions. Across ten studies comprising 108 patients, the combined use of fluorescence guidance and functional mapping appears not only feasible and functionally safe, but also clinically useful in supporting maximal safe resection. Gross total resection was achieved in 57.4% overall and in 66.7% of patients in the largest cohort, while permanent morbidity remained within the expected range for eloquent glioma surgery. Although the evidence base remains largely observational, the consistency of the reported intraoperative utility of 5-ALA across case reports, case series, and cohort data supports its role as a valuable adjunct—particularly in high-grade tumors where fluorescence positivity is much more frequent.

### 4.1. Oncologic Impact of 5-ALA in the Awake Setting

The oncologic benefit of 5-ALA in glioma surgery has been well established in the general anesthesia setting [50,51,52,53,54,55]. The landmark randomized controlled trial by Stummer et al. [56] demonstrated improved rates of complete resection of contrast-enhancing tumor and progression-free survival in glioblastoma when 5-ALA was used. Subsequent meta-analyses have confirmed its high sensitivity for high-grade glioma (HGG), particularly WHO Grade IV tumors [24].

Interpretation of fluorescence findings must also account for the substantial molecular heterogeneity that exists within histologically defined glioma grades. Gliomas of similar WHO grade can differ markedly in IDH mutation status, proliferative capacity, and metabolic phenotype, and these molecular differences plausibly influence both PpIX accumulation and clinical behavior independently of histological grade [57,58]. In the present review, IDH status was reported in only four of ten included studies, precluding a formal molecular subgroup analysis of fluorescence behavior. This is an important limitation, as a tumor classified as “high-grade” on histology alone may represent a biologically heterogeneous mixture of IDH-mutant and IDH-wildtype disease, with correspondingly variable fluorescence and prognosis. Future studies integrating 5-ALA fluorescence data with molecular classification are needed to determine whether fluorescence behavior should be interpreted within, rather than across, molecularly defined subgroups [57,58].

5-ALA is not the only fluorescent agent used in glioma surgery. Sodium fluorescein, a non-specific blood–brain barrier permeability marker, is used as a lower-cost alternative or adjunct that highlights regions of barrier disruption rather than tumor metabolic activity directly, and several comparative series suggest broadly similar rates of resection benefit between the two agents, albeit with different fluorescence mechanisms, specificity profiles, and visualization equipment requirements [27]. None of the studies included in this review used sodium fluorescein or other fluorescent dyes in combination with or as an alternative to 5-ALA during AC, and a direct comparison of agent-specific performance in the awake setting therefore remains an open question for future study.

Applying the WHO classification retrospectively to this literature also illustrates a specific interpretive limitation. Haider et al. [44] report a Grade III, IDH-wildtype diffuse astrocytoma. Under WHO 2021 criteria, an IDH-wildtype astrocytic tumor harboring any of the three recognized molecular markers of glioblastoma-level aggressiveness (TERT promoter mutation, EGFR amplification, or combined whole-chromosome 7 gain/10 loss) is classified as glioblastoma, IDH-wildtype, CNS WHO grade 4, regardless of histological grade on light microscopy. TERT promoter, EGFR, and chromosome 7/10 status were not reported in Haider et al. [44], and it is therefore not possible to determine from the published source whether this tumor would be molecularly upgraded to glioblastoma under current criteria. This example illustrates a broader limitation of the present evidence base, because most included studies were published before or around the 2021 WHO revision and do not uniformly report the relevant molecular markers. Therefore, grade- and diagnosis-stratified analyses in this review should be interpreted as reflecting the classification criteria in use at the time each study was conducted, which may not be fully concordant with current WHO CNS5 diagnostic categories.

Our findings extend the established oncologic utility of 5-ALA into the awake setting. Fluorescence positivity was observed in 76.3% of high-grade gliomas, closely mirroring the wider fluorescence-guided surgery literature and supporting the reliability of 5-ALA even when surgery is performed under awake conditions. Bright fluorescence was seen predominantly in Grade IV tumors, consistent with, but not confirmatory of, a biological association between protoporphyrin IX accumulation and aggressive tumor metabolism [59,60]. This association should be interpreted cautiously, as fluorescence intensity is influenced by multiple factors beyond tumor grade, including tumor cellularity, blood–brain barrier integrity, necrosis, 5-ALA dose and timing, and intraoperative optical conditions, none of which were systematically controlled or reported across the included studies. The present data are therefore consistent with, but cannot independently establish, a grade-dependent biological gradient in PpIX metabolism. In the studies reporting tissue correlation, fluorescence–histology concordance was high, indicating that 5-ALA remains a dependable intraoperative marker of tumor tissue even when the surgeon must work within functionally constrained margins [44,45,47].

Although fluorescence was less frequent in low-grade gliomas (17.1%), this does not diminish the overall value of 5-ALA in awake glioma surgery; rather, it helps define where its utility is greatest. The present evidence most strongly supports use in high-grade and contrast-enhancing lesions, where fluorescence can meaningfully improve detection of residual tumor and assist resection strategy. In lower-grade lesions, its role is more selective and adjunctive.

A further limitation of the functional outcome evidence base is its near-exclusive reliance on aphasia and hemiparesis as the reported endpoints. Only two included studies assessed broader domains: Drosos et al. [42] incorporated multitasking and Stroop testing, and Haider et al. [44] documented cognitive difficulty, neglect, and acalculia as baseline deficits. No included study used a standardized multimodal neurocognitive battery, and fatigue, processing speed, and intraoperative task performance were not systematically quantified in any report. More objective, multimodal approaches to intraoperative and postoperative functional monitoring, combining task-based assessment with quantitative or physiological correlates, have been proposed to capture this broader functional landscape more reliably than binary deficit/no-deficit reporting [61]. Incorporating such multimodal assessment into future studies using 5-ALA in AC would substantially strengthen the precision with which functional safety can be defined and measured.

It is important to distinguish the oncologic surrogate outcomes reported here from demonstrated oncologic benefit. The present review relies predominantly on extent-of-resection data; recurrence status was reported for only 22 of 108 patients (20.4%), and follow-up duration across studies ranged from approximately 3 to 15 months, which is insufficient to characterize progression-free or overall survival in either high- or low-grade glioma. While improved intraoperative visualization and extent of resection are plausible mechanistic contributors to oncologic outcome based on the broader 5-ALA literature under general anesthesia [23,24,56], the present evidence supports 5-ALA’s contribution to surgical visualization and resection extent specifically within the awake setting, rather than a demonstrated survival benefit attributable to 5-ALA use during AC.

### 4.2. Functional Boundaries Versus Fluorescent Boundaries

One of the most important messages from this review is not that fluorescence and mapping are in conflict, but that they answer different intraoperative questions. Awake mapping defines what must be preserved; 5-ALA highlights where biologically active tumor remains. When the two boundaries diverge, the surgeon gains richer decision-making information rather than a contradiction. In several studies, resection was appropriately halted despite persistent fluorescence when stimulation identified essential language or motor pathways [43,48]. This should not be interpreted as a limitation of 5-ALA, but rather as evidence that fluorescence can successfully reveal residual tumor even when that tissue cannot be safely removed.

This distinction is biologically and surgically meaningful [62,63,64]. Fluorescence reflects altered tumor metabolism, whereas awake mapping identifies indispensable cortico-subcortical networks [65]. Because infiltrative gliomas may extend into or alongside eloquent pathways without immediately abolishing function, discordance between fluorescent and functional borders is expected rather than problematic [66,67]. In practice, this makes 5-ALA a particularly valuable complement to awake surgery: it enhances tumor conspicuity, facilitates targeted biopsy and selective resection in non-eloquent fluorescent regions, and helps the surgeon recognize when residual disease persists at a functionally protected margin [25,68,69,70]. The largest comparative study [48] supports this interpretation, showing that a “5-ALA-free” cavity was associated with gross total resection, while mapping remained the decisive safeguard of function.

The interpretation of postoperative neurological deficits in this literature has also been limited by an almost exclusive focus on direct mechanical or electrophysiological injury to eloquent cortical or subcortical structures as the presumed mechanism. None of the included studies systematically considered or excluded secondary injury mechanisms, including oxidative stress, mitochondrial dysfunction, peritumoral neuroinflammation, or apoptotic cascades, that may contribute independently to postoperative neurological change, whether related to tumor resection generally or, speculatively, to photodynamic or metabolic effects of 5-ALA/PpIX accumulation in peritumoral tissue [71]. Because no included study incorporated biomarkers or imaging correlates capable of distinguishing these mechanisms from mapping-related injury, the attribution of postoperative deficits in this review to functional mapping events alone should be regarded as the most parsimonious, but not the only possible, explanation.

### 4.3. Extent of Resection and Comparison to Awake Literature

The pooled GTR rate of 57.4% overall, and 66.7% in the largest cohort, compares favorably with published awake surgery series in eloquent gliomas [8,14,15]. Importantly, these resection rates were achieved in anatomically and functionally challenging tumors while maintaining permanent neurological morbidity within accepted expectations for eloquent glioma surgery. This pattern supports the practical value of 5-ALA in helping surgeons pursue maximal safe resection under awake conditions.

The benefit of 5-ALA in this setting should not be judged solely by whether pooled GTR percentages dramatically exceed historical awake series benchmarks [8,14,15]. In eloquent tumors, the final stopping point is intentionally governed by functional mapping, so the full theoretical cytoreductive advantage of fluorescence may be constrained by design [14,15,43,48]. Even so, 5-ALA appears to provide meaningful operative benefit by improving visualization at the tumor–brain interface, increasing confidence in resection of non-eloquent fluorescent tissue, helping identify residual tumor that may otherwise be overlooked under white light, and offering an immediate metabolic correlate to the surgeon’s anatomical and electrophysiological findings [16,17,43,44,48]. In essence, 5-ALA empowers the surgeon to make an informed choice on safety of resection within constraints of eloquence.

This interpretation is supported by studies in the present review in which residual fluorescent foci were specifically identified and resected, as well as by the association between a “5-ALA-free” cavity and gross total resection in the largest cohort [41,42,43,48]. Viewed in this way, fluorescence does not need to override mapping to be valuable; rather, it strengthens the surgeon’s ability to maximize resection up to the functional limit [43,48]. Taken together, the current evidence supports the selective use of 5-ALA particularly in high-grade, contrast-enhancing, or otherwise complex eloquent gliomas, where the added visual information is most likely to influence operative decision-making [16,17,43,44,48].

Notably, no study reported conversion to general anesthesia. Although publication bias cannot be excluded, the available data suggest that incorporation of fluorescence guidance does not materially disrupt the workflow of awake surgery when used in experienced hands.

The transient aphasia rate of 32.6% and persistent hemiparesis rate of 16.9% observed in this review fall within rates reported in the established AC literature for eloquent-region glioma surgery generally [72,73,74]. However, because none of the included studies incorporated a matched comparator group undergoing awake mapping without 5-ALA, the present data cannot establish whether 5-ALA independently influences neurological morbidity, whether favorably, unfavorably, or neutrally. Historical and cross-study comparisons of this kind are vulnerable to confounding by tumor grade, eloquent location, mapping expertise, patient selection, and concurrent adjunct use, any of which could account for the observed morbidity rates independently of 5-ALA. The most appropriate interpretation of the available evidence is therefore that 5-ALA-guided resection during AC can be performed with permanent morbidity rates broadly comparable to awake eloquent-region glioma surgery generally, rather than that 5-ALA has been shown not to increase neurological morbidity. This more cautious framing is concordant with the broader glioma literature, in which 5-ALA-guided resection is associated with improved extent of resection without clearly demonstrated excess morbidity, but typically also without a rigorously matched comparator [16,17].

### 4.4. Timing and Technical Considerations

Most studies used the conventional 20 mg/kg dosing regimen administered 2–3 h preoperatively, indicating that standard 5-ALA workflows are readily transferable to AC. Established pharmacokinetic data indicate that peak fluorescence occurs approximately 6–8 h after oral administration [56]. Against this background, Haider et al. [44] reported a single case in which clinically useful fluorescence was still present at an atypical interval of approximately 32–36 h after administration. This single observation should not be generalized to characterize the reliability of fluorescence beyond the conventional 2–4 h administration window; it represents a hypothesis-generating outlier rather than evidence that the fluorescence window is routinely more forgiving than established pharmacokinetics suggest, and standard dosing intervals should continue to guide clinical practice pending confirmatory data from additional cases or prospective timing studies.

Awake fluorescence-guided surgery does introduce specific technical considerations, including low-light conditions during patient interaction and the need to coordinate blue-light visualization with real-time language or motor testing [43,44,45,46,47]. Nevertheless, the available reports show that these challenges are manageable, and no study described abandonment of fluorescence because of patient intolerance. Taken together, this supports the view that 5-ALA can be integrated into awake workflows without major practical compromise [43,44,45,46,47,48].

### 4.5. Integration with Intraoperative MRI and Adjuncts

In the only comparative study, adding intraoperative MRI to 5-ALA did not significantly change GTR rates, with both groups achieving 66.7% gross total resection. Rather than diminishing the value of fluorescence, this finding suggests that 5-ALA alone already provides meaningful operative guidance in many awake glioma cases. At the same time, fluorescence and iMRI appear to provide different but potentially synergistic information: fluorescence reflects metabolically active tissue at the operative surface, whereas iMRI identifies structural residual disease that may not be visibly fluorescent. Multimodal integration may therefore be best viewed as an escalation of guidance for selected complex tumors rather than a replacement for 5-ALA.

### 4.6. Future Directions

Future studies should now move beyond feasibility and focus on defining where 5-ALA adds the greatest value within awake glioma surgery, including in combination with complementary preoperative planning tools such as nTMS mapping, which allows non-invasive preoperative localization of eloquent cortex and could be used to inform both patient selection for 5-ALA-guided AC and the intraoperative testing strategy applied once fluorescence-guided resection begins [28,29]. Prospective multicenter studies using standardized volumetric extent-of-resection metrics, survival outcomes, and structured neurocognitive follow-up would help quantify the magnitude of benefit and refine patient selection. Comparative cohort designs evaluating awake mapping alone versus mapping combined with 5-ALA would be especially valuable, not because the utility of fluorescence is in doubt, but because its contribution should now be measured more precisely across tumor grades, locations, and operative strategies. Standardization of fluorescence grading, incorporation of quantitative spectroscopy, and integration of molecular profiling may further strengthen the precision of fluorescence-guided onco-functional surgery.

Technological convergence offers a transformative opportunity for the field. Augmented reality (AR) platforms capable of overlaying fluorescence maps, tractography, and real-time functional mapping data directly into the operative field could reduce cognitive load and improve spatial precision when oncologic and functional boundaries diverge [75,76,77]. Virtual reality (VR)-based preoperative simulation may further enhance surgical planning in complex eloquent tumors, allowing rehearsal of anticipated fluorescence–function conflicts [75,76]. Coupled with artificial intelligence-driven predictive modeling of safe resection margins, these innovations have the potential to create an integrated, data-driven operative ecosystem in which tumor visualization and network preservation are dynamically balanced [77,78,79,80,81]. Prospective evaluation of these technologies will determine whether digital augmentation can meaningfully improve both oncologic and functional outcomes in precision neuro-oncology.

### 4.7. Limitations

Several limitations must be acknowledged. First, this review was not prospectively registered in a public registry (e.g., PROSPERO); although eligibility criteria and outcome domains were fixed internally before screening, the absence of public registration limits external verification of a priori decisions and should be considered when weighing the certainty of our conclusion. Second, the overall evidence base is predominantly composed of case reports and retrospective observational studies. Seven of ten included studies were single-patient reports, inherently subject to selection and publication bias. Positive or technically successful cases are more likely to be published, potentially overestimating safety and oncologic efficacy. Third, there was substantial heterogeneity in study design, reporting standards, tumor grade distribution, adjunct usage, and outcome definitions. Extent of resection was not uniformly assessed using volumetric analysis, and long-term survival data were limited. More specifically, different paradigms have been used to estimate extent of resection based on T1 enhancing regions vs. T2 FLAIR hyperintensities which makes pooling the extent of resection data less accurate. Further studies with precise uniform definition of extent of resection is required to evaluate the role of 5-ALA in increasing the extent of resection. Fourth, follow-up duration was inconsistently reported. Only a subset of patients had documented medium-term imaging or survival outcomes. Therefore, pooled recurrence and survival estimates should not be interpreted as representative incidence rates. Fifth, the timing between administration of 5-ALA and tumor resection is not reported in some of the studies or it is reported beyond the time interval of fluorescence peak which can affect the efficacy of 5-ALA in showing tumor cells. Sixth, no randomized controlled trials or prospective comparative studies were identified. Consequently, the precise magnitude of the added benefit conferred by 5-ALA within the awake setting, beyond what is achieved with mapping alone, cannot yet be quantified with high certainty. Finally, fluorescence detection thresholds and intraoperative interpretation were not standardized across studies. Variability in microscope technology, surgeon experience, and subjective interpretation may influence reported fluorescence rates.

## 5. Conclusions

5-ALA-guided resection during AC is a feasible and clinically valuable adjunct for glioma surgery in eloquent regions. The available evidence supports its ability to enhance intraoperative tumor visualization, assist identification of residual disease, and facilitate maximal safe resection with permanent neurological morbidity rates broadly comparable to those reported for awake eloquent-region glioma surgery generally. In the absence of a matched non-5-ALA comparator, however, these data do not establish whether 5-ALA independently alters neurological morbidity relative to awake mapping alone. Functional mapping remains the decisive determinant of the final resection boundary, but this does not diminish the contribution of fluorescence; rather, the two modalities appear complementary and together support an onco-functional surgical strategy. Prospective, standardized, multicenter studies are warranted to quantify the magnitude of benefit, optimize patient selection, and clarify long-term oncologic impact.

## Figures and Tables

**Table 1:** A summary of study characteristics.

Study	Title	Country	Study Design	Sample Size	Study Period	Inclusion/Exclusion Criteria
**Corns et al., 2015** [39]	5-aminolevulinic acid guidance during awake craniotomy to maximize extent of safe resection of glioblastoma multiforme	United Kingdom	Single case report	1 patient	Initial surgery July 2012; recurrence 16 months later	Recurrent left frontal GBM encroaching on Broca’s area; candidate for AC and 5-ALA
**Skjøth-Rasmussen et al., 2017** [40]	Awake craniotomy with 5-ALA in high grade gliomas gives simultaneous guidance of tumor and functional border	Denmark	Retrospective case series	19 patients	Not reported	Suspected HGG undergoing combined 5-ALA-assisted surgery and AC
**Brosnan et al., 2022** [41]	Anesthetic Selection for an Awake Craniotomy for a Glioma with Wernicke’s Aphasia	United States	Single case report	1 patient	2022	High-grade glioma near Wernicke’s area undergoing SAS awake AC with 5-ALA
**Drosos et al., 2022** [42]	Awake brain surgery for autistic patients: Is it possible?	United Kingdom	Single case report	1 patient	2022	To present a case of a patient with autistic spectrum disorder who was successfully operated on for a brain tumor through AC
**Goryaynov et al., 2022** [43]	5-ALA-guided tumor resection during awake speech mapping in gliomas located in eloquent speech areas	Russia	Prospective single-center observational study	40 patients	2008–2020	Patients age over 18 years old, and diagnosis of left frontal or temporal glioma requiring awake surgery with intraoperative detection of the eloquent speech cortical areas and tracts, including superior longitudinal fasciculus, arcuate fasciculus, or frontal aslant tract
**Haider et al., 2022** [44]	Clinically useful tumor fluorescence greater than 24 h after 5-aminolevulinic acid administration	United States	Single case report	1 patient	Not specified	Newly diagnosed dominant hemisphere high-grade glioma planned for AC with 5-ALA
**Price et al., 2023** [45]	Awake resection of recurrent astroblastoma with intraoperative 5-ALA–induced fluorescence: illustrative case	United States	Single case report	1 patient	Recurrence 21 years after initial diagnosis; index surgery age 32	Recurrent astroblastoma in dominant hemisphere undergoing AC with 5-ALA
**Matei et al., 2024** [46]	Awake surgery during 5-ALA high grade glioma fluorescence guided resection	Romania	Single case report	1 patient	Surgery performed May 2023	Frontal high-grade glioma requiring AC with 5-ALA
**Moldabekov et al., 2024** [47]	Awake magic: glioblastoma resection under 5-aminolevulinic acid guidance during awake craniotomy. A case report with video demonstration	Kazakhstan	Single case report	1 patient	Surgery performed June 2022	Contrast-enhancing left frontotemporal glioma involving Broca’s area requiring awake mapping + 5-ALA
**Tagle-Vega et al., 2025** [48]	Integration of 5-ALA fluorescence and intraoperative MRI in awake craniotomy for glioma resection	Brazil	Retrospective single-center cohort study	42 patients	2016–2022	To analyze the integration of 5-ALA fluorescence-guided surgery and iMRI during AC for glioma resection at a single center in Brazil

Abbreviations: AC, awake craniotomy; ALA, aminolevulinic acid; GBM, glioblastoma multiforme; HGG, high-grade glioma; iMRI, intraoperative magnetic resonance imaging; SAS, sleep-awake-sleep.

**Table 2:** (**a**) Joanna Briggs Institute (JBI) critical appraisal summary of included studies with overall risk-of-bias classification. (**b**) Worked item-level JBI appraisal—Corns et al., 2015 [39] (8-item JBI Case Report Checklist).

(a)					
Study	Yes	No	Unclear	Quality Score (%)	Overall Risk of Bias
**Corns et al., 2015 (Case Report)** [39]	7	0	1	87.5%	Low
**Skjøth-Rasmussen et al., 2017 (Case Series—Abstract)** [40]	2	0	8	20.0%	High
**Brosnan et al., 2022 (Case Report)** [41]	7	0	1	87.5%	Low
**Drosos et al., 2022 (Case Report)** [42]	8	0	0	100.0%	Low
**Goryaynov et al., 2022 (Cohort Study)** [43]	6	1	4	54.5%	Moderate
**Haider et al., 2022 (Case Report)** [44]	7	0	1	87.5%	Low
**Price et al., 2023 (Case Report)** [45]	8	0	0	100.0%	Low
**Matei et al., 2024 (Case Report)** [46]	4	0	4	50.0%	Moderate
**Moldabekov et al., 2024 (Case Report)** [47]	7	0	1	87.5%	Low
**Tagle-Vega et al., 2025 (Cohort Study)** [48]	7	1	3	63.6%	Moderate
**(b)**					
**Item**	**Judgement**		**Rationale**		
1. Patient demographics clearly described	Yes		Age, sex, tumor history, and prior treatment reported		
2. Patient history clearly described, presented as timeline	Yes		Prior resection and chemoradiotherapy timeline reported		
3. Current clinical condition clearly described	Yes		Preoperative word-finding difficulty documented		
4. Diagnostic tests/assessment methods and results clearly described	Yes		Preoperative/postoperative MRI described		
5. Intervention(s)/treatment procedure(s) clearly described	Yes		AC, 5-ALA dose/timing, and mapping protocol described		
6. Post-intervention clinical condition clearly described	Yes		Postoperative speech status and pseudomeningocele reported		
7. Adverse events/unanticipated events identified and described	Yes		Transient pseudomeningocele requiring lumbar drainage reported		
8. Does the case report provide takeaway lessons?	Yes		Authors’ conclusion on feasibility clearly mentioned		

Case reports were assessed using the 8-item JBI Case Report Checklist; the case series was assessed using the 10-item JBI Case Series Checklist; cohort studies were assessed using the 11-item JBI Cohort Checklist. Quality score (%) represents the proportion of criteria rated “Yes” relative to total checklist items. Overall risk of bias classification: Low (≥80%), Moderate (50–79%), High (<50%). Overall risk of bias: Low (8/8 “Yes”). This worked example illustrates the item-level approach; the corresponding item-level appraisal for the remaining nine studies, reflecting the original consensus judgement of the two independent raters (M.M. and M.S.M.).

**Table 3:** An Overview of Patient characteristics.

Study	Age (Mean, Range)	Sex	Handedness	Preoperative Karnofsky Performance Status (KPS)	Preoperative Neurological Deficits: Language, Motor, Cognitive/Neuropsychological	Special Populations (e.g., ASD, Elderly, Epilepsy History, Comorbidities)
**Corns et al., 2015** [39]	32 years	Female: 1 (100%); Male: 0 (0%)	Right-handed: 1 (100%)	Not reported	Subtle word-finding difficulties: 1 (100%)	Recurrent GBM; prior Stupp protocol chemoradiotherapy: 1 (100%)
**Skjøth-Rasmussen et al., 2017** [40]	Median 54 years (range 35–70)	Female: 9 (47.4%); Male: 10 (52.6%)	Not reported	Not reported	Not reported	Reoperations: 9/19 (47.4%) were undergoing ≥ second surgery (5 s surgery [26.3%]; 4 third or more [21.1%])
**Brosnan et al., 2022** [41]	73 years	Female: 1 (100%)	Left language dominance on fMRI	Not reported	Profound naming and word-finding difficulty (Wernicke’s aphasia); short-term memory loss: 1 (100%)	Elderly (≥70): 1 (100%); comorbidities: diabetes, hypertension, anxiety, Hashimoto’s thyroiditis, osteoarthritis, lumbar spinal stenosis
**Drosos et al., 2022** [42]	42 years	Male: 1 (100%);	Right-handed: 1 (100%)	Not reported	Mild speech hesitation, seizures	Autism spectrum disorder diagnosed in childhood; learning difficulties; anxiety features; dependent on primary caregiver
**Goryaynov et al., 2022** [43]	Not reported	Not reported	Assessed via Annett questionnaire (numbers not reported)	Not reported	Variable, including aphasia	Not reported
**Haider et al., 2022** [44]	66 years	Male: 1 (100%)	Right-handed: 1 (100%)	Not reported	Mild cognitive difficulty, dysphasia, gait difficulty, subtle neglect, acalculia, extinction to double simultaneous stimuli	Dominant hemisphere tumor
**Price et al., 2023** [45]	32 years	Female: 1 (100%)	Right-handed: 1 (100%)	Not reported	Generalized tonic-clonic seizure, language and motor deficit: None reported preoperatively	Bipolar disorder; intractable migraines, multiple prior resections (three prior surgeries); prior radiation therapy
**Matei et al., 2024** [46]	Not reported	Not reported	Not reported	Not reported	Not reported	Not reported
**Moldabekov et al., 2024** [47]	55 years	Female: 1 (100%)	Right-handed: 1 (100%)	70	Disorientation (space/time); reduced executive function; central right facial paresis (HB grade 3); right tongue deviation; mild-moderate dynamic aphasia	Dominant hemisphere GBM
**Tagle-Vega et al., 2025** [48]	42–50 years	Male: 30 (71.4%); Female: 12 (28.6%)	Not reported	Median 90	Aphasia, seizures, motor deficits	Not reported

Abbreviations: ASD, autism spectrum disorder; GBM, glioblastoma multiforme; HB, House–Brackmann.

**Table 4:** An overview of tumor characteristics.

Study	Tumor Type	WHO Grade (II–IV)	Molecular Markers	Tumor Location	Contrast-Enhancing vs. Non-Enhancing	Tumor Volume
**Corns et al., 2015** [39]	GBM (recurrent)	Grade IV	Not reported	Left frontal lobe encroaching on Broca’s area	T1 contrast-enhancing lesion present	Not reported
**Skjøth-Rasmussen et al., 2017** [40]	Suspected high-grade glioma (19, 100%)	Not reported	Not reported	Close relationship with eloquent structures (100%)	Not reported	Not reported
**Brosnan et al., 2022** [41]	Glioma (histology confirmed GBM)	Grade IV	Not reported	Left temporal lobe, adjacent to Wernicke’s area and arcuate fasciculus	Heterogeneously enhancing 4 cm mass	4 cm mass with vasogenic edema and 3–4 mm midline shift
**Drosos et al., 2022** [42]	Diffuse astrocytoma	Grade: III	IDH mutant	Left superior frontal gyrus (mid-posterior), involving SMA, extension to cingulate gyrus	Non-contrast-enhancing tumor	26.8 cm^3^
**Goryaynov et al., 2022** [43]	Diffuse gliomas	Grades: II (n = 10, 36%), III (n = 6, 21%), IV (n = 12, 43%)	Not reported	Left frontal or temporal, eloquent speech areas (Broca, Wernicke, AF, SLF, FAT)	Mixed (HGG mostly enhancing)	Not reported
**Haider et al., 2022** [44]	Diffuse astrocytoma	Grade III	IDH wild-type	Left supramarginal gyrus (dominant parietal lobe)	Partially cystic contrast-enhancing mass	Not reported
**Price et al., 2023** [45]	Astroblastoma	Astroblastoma, MN1-altered	In-frame *MN1::BEND2* fusion	Left parietal region (anterior inferior aspect of prior resection cavity)	Strongly enhancing lesion	3.8 × 2.9 × 3.0 cm
**Matei et al., 2024** [46]	High-grade glioma	Not reported	Not reported	Frontal lobe (language area)	Not reported	Not reported
**Moldabekov et al., 2024** [47]	Glioblastoma	Grade IV	IDH wild-type	Left frontotemporal region involving Broca’s area	Enhancing lesion	Not reported
**Tagle-Vega et al., 2025** [48]	LGG 40–47%, HGG 53–59%	Grades: II (n = 18, 42.9%), III (n = 6, 14.3%), IV (n = 18, 42.9%)	Not reported	Majority in eloquent areas (67–85%)	Not reported	Not reported

Abbreviations: AF, arcuate fasciculus; FAT, frontal aslant tract; HGG, high-grade glioma; IDH, isocitrate dehydrogenase; IV, intravenous; LGG, low-grade glioma; SLF, superior longitudinal fasciculus; SMA, supplementary motor area.

**Table 5:** Awake craniotomy protocols.

Study	Awake Craniotomy Technique	Anesthetic Agents	Patient Positioning and Fixation	Mapping	Intraoperative Testing	Intraoperative Complications
**Corns et al., 2015** [39]	Sleep-awake technique: initial GA for craniotomy and partial debulking, then awake for mapping and resection	General anesthesia (agents not specified); dexamethasone preoperatively	Not reported	Cortical speech mapping	Expressive speech monitored by speech and language therapist	None
**Skjøth-Rasmussen et al., 2017** [40]	Not reported	Not reported	Not reported	Awake functional mapping (100%)	Not reported	Not reported
**Brosnan et al., 2022** [41]	Sleep-awake-sleep	Propofol infusion (100–150 mcg/kg/min), remifentanil (0.02–0.03 mcg/kg/min), dexmedetomidine (0.2 mcg/kg/h), propofol boluses	Not reported	Awake cortical speech mapping	Continuous object naming, speech arrest testing, conversation	Fatigue and intermittent somnolence during awake phase; no neurological deterioration
**Drosos et al., 2022** [42]	Awake throughout	Dexmedetomidine	Supine, head rotated 30° right, horseshoe headrest (no pin fixation)	Motor and language	Motor tasks, nine-peg test, speech (counting, free speech, Stroop), multitasking	Single focal seizure (controlled with cold saline); fully cooperative throughout (100%)
**Goryaynov et al., 2022** [43]	Sleep-awake-sleep	Not reported	Not reported	Bipolar cortical stimulation; monopolar subcortical stimulation	Continuous neuropsychological speech assessment	None
**Haider et al., 2022** [44]	Not reported	Not reported	Not reported	Cortical mapping; subcortical motor-evoked potentials; phase reversal	Not reported	Preoperative fever (surgery delayed); no intraoperative complications
**Price et al., 2023** [45]	Not reported	Not reported	Not reported	Direct cortical stimulation for speech mapping	Not reported	Not reported
**Matei et al., 2024** [46]	Sleep-awake-sleep	Not reported	Not reported	Direct cortical stimulation (Penfield bipolar technique)	Boston naming test; calculation tasks; structured language tasks	Not reported
**Moldabekov et al., 2024** [47]	Sleep-awake-sleep	Dexmedetomidine (200 mg total reported); fentanyl 0.2 mg	Not reported	Not reported	Broca area language mapping; motor tasks (right hand/leg movement)	Not reported
**Tagle-Vega et al., 2025** [48]	Sleep–awake–sleep	Not reported	Not reported	Direct cortical stimulation in 100%	Language, motor, cognition	Not reported

**Table 6:** 5-ALA-specific variables.

Study	Dose and Timing of 5-ALA Administration	Fluorescence Detection Method	Presence and Rate of Fluorescence	Fluorescence–Histology Concordance	Use of 5-ALA in: Cortical Phase, Subcortical Phase, Only Non-Eloquent Regions	Reported Limitations of 5-ALA
**Corns et al., 2015** [39]	1.5 g orally (single dose), 6 h prior to induction of GA	Intense fluorescence in tumor core with subtle pink fluorescence at tumor margins	Present: 1 (100%), intense fluorescence core + subtle pink border zone	Fluorescing tumor was remote from speech cortex; resection guided by awake mapping at eloquent boundary	Yes (cortical); yes (non-cortical, guided extended resection)	Not reported
**Skjøth-Rasmussen et al., 2017** [40]	Not reported	Not reported	Not reported	Not reported	Yes (cortical); subcortical implied but not specified	Not reported
**Brosnan et al., 2022** [41]	5-ALA use: administered preoperatively (Gliolan)	Fluorescence-guided resection used in conjunction with stereotactic navigation	Used to guide deeper tumor resection	Not reported	Cortical yes; subcortical yes—allowed continued resection when mapping limited by fatigue	Not reported
**Drosos et al., 2022** [42]	Not reported	Blue light visualization intraoperatively	Used to identify residual tumor in non-eloquent cavity regions	Not reported	Used as adjunct for non-eloquent residual tumor	Not reported
**Goryaynov et al., 2022** [43]	Dose: 20 mg/kg orally, 2 h preop	Zeiss BLUE400	Fluorescence present in 20 of 34 5-ALA-treated patients (58.8%): early cohort (n = 28, cortical mapping only)—fluorescence present in 14 (50.0%; bright in 10 [all Grade IV], weak in 4), absent in 14; later cohort (n = 12, cortical and subcortical mapping), of whom 6 received 5-ALA and 6 did not—all 6 5-ALA-treated patients in this cohort showed fluorescence (4 Grade IV, 1 Grade III, 1 Grade II); the 6 patients who did not receive 5-ALA are excluded from this analysis. By grade across both 5-ALA-treated subgroups (n = 34): Grade IV fluorescent in 14/16 (87.5%), Grade III fluorescent in 2/7 (28.6%), Grade II fluorescent in 4/11 (36.4%)	Not reported	Fluorescence used to guide biopsy and resection	Not reported
**Haider et al., 2022** [44]	20 mg/kg orally, given 5:50 AM; surgery performed ~32–36 h later	Blue-light microscopy	Yes, clinically useful fluorescence present at 32–36 h	All fluorescent specimens positive for tumor	Yes (cortical and subcortical)	Prolonged interval beyond recommended 2–4 h window; concern regarding photobleaching and metabolic clearance
**Price et al., 2023** [45]	Not reported	Blue-light fluorescence during surgery	Yes (heterogeneous fluorescence)	Fluorescent tumor confirmed histologically	Yes (cortical), implied (subcortical)	Not reported
**Matei et al., 2024** [46]	Not reported	Blue-light fluorescence during surgery	Yes	Not reported	Yes (cortical and subcortical)	Requirement for low-light conditions due to photosensitization risk
**Moldabekov et al., 2024** [47]	Not reported	BLUE400 fluorescence microscopy	Yes (intense residual fluorescence in Broca region)	Fluorescent areas confirmed GBM	Yes (cortical and subcortical)	Conflict between fluorescence persistence and functional preservation
**Tagle-Vega et al., 2025** [48]	20 mg/kg, 3 h preop	Not reported	Not reported	Not reported	Fluorescence strongly associated with high-grade tumors (*p* < 0.001)	Not reported

Abbreviations: GA: General anesthesia; GBM: Glioblastoma multiforme.

**Table 7:** Adjuncts and extent of resection.

Study	Adjuncts Used Alongside 5-ALA	Extent of Resection	Method of Assessment	Residual Tumor Location	Whether 5-ALA Directly Influenced Stopping Points
**Corns et al., 2015** [39]	Functional MRI (preoperative); awake cortical mapping; carmustine (BCNU) wafers placed intraoperatively	GTR of all T1-contrast-enhancing tumor	Postoperative contrast-enhanced MRI	Partial residual T2/FLAIR signal left intentionally	Yes—resection included intense and subtle fluorescence but preserved normal brain tissue
**Skjøth-Rasmussen et al., 2017** [40]	Not reported	Complete or near-complete resection (10/19, 52.6%); >90% tumor volume resection (12/19, 63.2%); first-time GBM subgroup GTR (6/9; 66.7%)	Postoperative MRI with volumetric measurement	Not reported	Implied yes (tumor + functional border guidance)
**Brosnan et al., 2022** [41]	Neuronavigation; bispectral index (BIS) monitoring; functional MRI (preoperative)	~95% tumor resection	Surgeon estimate (postoperative imaging implied but not quantified)	Deep borders (not fully detailed)	Yes—allowed continued resection when patient became fatigued
**Drosos et al., 2022** [42]	Intraoperative ultrasound (iUS); frameless stereotactic navigation; IOM; ECoG	Resection: 81%	Postoperative MRI volumetric measurement (26.8 cm^3^ → 5.2 cm^3^)	Posterolateral margin near corticospinal tract. Residual left due to functional boundaries	Yes—used to resect enhancing tumor in non-eloquent regions
**Goryaynov et al., 2022** [43]	Neuronavigation; tractography; neurophysiological monitoring	Early cohort (n = 28): postoperative imaging available for 18/28 (64%) at 48 h—GTR ^1^ in 12 (67%), subtotal in 3 (17%), partial in 3 (17%); extent of resection not available for the remaining 10/28. Later cohort, 5-ALA-treated patients only (n = 6): GTR achieved in all 6 (100%). Combined evaluable GTR among 5-ALA-treated patients with imaging available: 18/24 (75.0%)	MRI available within 48 h (18, 64%)	Residual fluorescence often intentionally left due to positive speech mapping	Yes—resection deliberately stopped in presence of fluorescence when speech disturbance occurred (60% of fluorescent cases).
**Haider et al., 2022** [44]	Awake cortical mapping; subcortical MEPs; intraoperative MRI	GTR of contrast-enhancing tumor	Postoperative MRI (POD2)	Non-enhancing T2 hyperintensity at superior margin	Yes—fluorescence used to guide resection despite delayed surgery
**Price et al., 2023** [45]	Awake speech mapping	GTR	Postoperative MRI (3 months confirmed GTR)	None on follow-up MRI	Yes—used to confirm complete resection (no residual fluorescence)
**Matei et al., 2024** [46]	DTI tractography; neuronavigation; intraoperative neurophysiological monitoring	GTR	Not reported	Not reported	Yes—used alongside mapping to achieve GTR
**Moldabekov et al., 2024** [47]	Awake language mapping; neuronavigation; DTI tractography	GTR of enhancing tumor (immediate postoperative imaging)	Postoperative MRI + CT	Slight residual tissue at 3-month MRI, stable	Yes—resection halted despite fluorescence when speech arrest occurred
**Tagle-Vega et al., 2025** [48]	Neuronavigation; direct cortical stimulation; intraoperative MRI (selected cases)	GTR: 66.7% in both groups	^1^ GTR is defined as residual tumor ≤ 0.175 cm^3^, however it is not cleared whether it is based on enhancing T1 or T2 FLAIR sequences	Not reported	5-ALA-free cavity strongly associated with GTR

Abbreviations: DTI, diffuse tensor imaging; ECoG, electrocorticography; GTR, gross total resection; IOM, intraoperative monitoring; MEP, motor-evoked potentials. ^1^ In this study, the extent of resection is defined based on comparing pre- and postoperative T1 contrast enhancement for glioblastomas or T2 FLAIR data for non-contrast-enhancing gliomas.

**Table 8:** Postoperative functional outcomes and follow-up across included studies.

Study	Functional Outcome	Follow-Up	Authors’ Conclusion
**Corns et al., 2015** [39]	No new or worsened speech deficit; transient pseudomeningocele requiring lumbar drainage	Immediate postoperative period	Combined use of AC and 5-ALA enables maximal safe resection in eloquent GBM without neurological deficit
**Skjøth-Rasmussen et al., 2017** [40]	No exacerbation of neurological condition	Not reported	Combined 5-ALA and awake mapping allows satisfactory tumor removal despite proximity to eloquent structures, maintaining onco-functional balance
**Brosnan et al., 2022** [41]	Repetition and naming intact; mild residual word-finding difficulty	Immediate postoperative course described	Careful anesthetic titration enables successful awake mapping even in patients with significant preoperative aphasia; minimum effective sedation recommended
**Drosos et al., 2022** [42]	No new postoperative deficits	Early postoperative and during chemoradiotherapy	AC with 5-ALA is feasible in carefully selected ASD patients. ASD is not an absolute contraindication to AC; with meticulous preparation, safe resection is feasible
**Goryaynov et al., 2022** [43]	Early cohort (n = 28): transient aphasia in 14 (50%), permanent aphasia in 4 (14%), hematoma requiring reoperation in 1 (3%). Later cohort (n = 12, both 5-ALA-treated and non-treated patients, as speech-mapping-related deficits are not 5-ALA-specific): transient aphasia in 11 (92%), permanent aphasia in 1 (8%). Combined across both cohorts (n = 40): transient aphasia in 25 (62.5%), permanent aphasia in 5 (12.5%)	None reported	5-ALA and awake speech mapping are complementary; allow maximal safe resection while preserving eloquent speech networks; fluorescence useful in HGG and prognostically informative in LGG
**Haider et al., 2022** [44]	No new focal deficits	Immediate postoperative period	Clinically useful fluorescence may persist >24 h after single 5-ALA dose; strict adherence to 2–4 h window may not be necessary
**Price et al., 2023** [45]	Returned to neurological baseline	3 months (MRI confirmed GTR)	5-ALA can assist in achieving GTR in astroblastoma; fluorescence may be strong despite rarity; maximizing resection critical for survival
**Matei et al., 2024** [46]	Language preserved	Not reported	Combining AC with 5-ALA fluorescence is safe and enables gross total resection while preserving language function
**Moldabekov et al., 2024** [47]	Speech and calculations improved, no permanent deficits	Not reported	5-ALA combined with AC maximizes safe GTR while preserving executive and motor function; multidisciplinary approach is essential
**Tagle-Vega et al., 2025** [48]	Persistent aphasia: 8/42 (19.0%) overall (3/15, 20.0%, in the “5-ALA + iMRI” subgroup; 5/27, 18.5%, in the ‘5-ALA only’ subgroup); transient aphasia: 4/42 (9.5%). Persistent hemiparesis: 14/42 (33.3%) overall (3/15, 20.0%, ‘5-ALA + iMRI’; 11/27, 40.7%, ‘5-ALA only’); transient paresis/hemiparesis: 5/42 (11.9%)	Not reported	Neurological sequelae significantly associated with eloquent location. 5-ALA improves intraoperative tumor delineation; iMRI provides complementary benefit, but functional mapping remains decisive

Abbreviations: AC: awake craniotomy; ALA: aminolevulinic acid; ASD, autism spectrum disorder; GBM, glioblastoma multiforme; GTR, gross total resection; HGG, high-grade glioma; LGG, low-grade glioma; MRI, magnetic resonance imaging.

**Table 9:** Headline outcomes stratified by study design.

Study Design	Studies (n)	Patients (n)	Fluorescence Positive	GTR	Persistent Aphasia	Persistent Hemiparesis
Single-patient case reports	7	7	6/6 evaluable (Price excluded, ungraded)	6/7 (85.7%)	0/7	0/7
Retrospective case series	1 (Skjøth-Rasmussen)	19	Not reported	10/19 (52.6%) complete/near-complete	Not separately reported	Not separately reported
Prospective/retrospective cohorts	2 (Goryaynov, Tagle-Vega)	82	44/76 evaluable (57.9%)	55/82 (67.1%) *	13/82 (15.9%)	15/82 (18.3%)
Overall (descriptive aggregate)	10	108	50/83 grade known (60.2%)	61/98 evaluable (62.2%)	13/89 (14.6%)	15/89 (16.9%)

* GTR denominator for cohorts reflects patients with evaluable postoperative imaging only. Abbreviations: GTR, gross total resection.

**Table 10:** Structured comparison of functional mapping protocols across included studies.

Study	Stimulation Type	Current/Frequency Parameters	Language/Motor Tasks Used	Subcortical Threshold/Stopping Criterion
Corns et al. [39]	Direct cortical stimulation	Not reported	Expressive speech, monitored by speech-language therapist	Resection stopped at onset of speech disturbance during monopolar stimulation
Skjøth-Rasmussen et al. [40]	Cortical functional mapping	Not reported	Not reported	Not reported
Brosnan et al. [41]	Cortical speech mapping	Not reported	Continuous object naming, speech arrest testing, conversation	Resection continued/modified based on fatigue-limited mapping reliability
Drosos et al. [42]	Cortical and subcortical (motor and language)	Not reported	Motor tasks, nine-peg test, counting, free speech, Stroop, multitasking	Resection stopped at positive subcortical corticospinal tract stimulation
Goryaynov et al. [43]	Bipolar cortical; monopolar subcortical	50–60 Hz, 3–4 mA (cortical)	Continuous neuropsychological speech assessment, computerized naming test	Resection stopped at stimulation-induced speech alteration, confirmed by bipolar re-stimulation
Haider et al. [44]	Cortical mapping; subcortical motor-evoked potentials; phase reversal	Not reported	Not reported	Not reported
Price et al. [45]	Direct cortical stimulation	Not reported	Speech mapping tasks (not detailed)	Not reported
Matei et al. [46]	Direct cortical stimulation (Penfield bipolar technique)	Not reported	Boston naming test, calculation tasks, structured language tasks	Not reported
Moldabekov et al. [47]	Not reported	Not reported	Broca-area language mapping; right hand/leg motor tasks	Resection halted at speech arrest despite residual fluorescence
Tagle-Vega et al. [48]	Direct cortical stimulation (100% of cases)	Not reported	Language, motor, cognition tasks (not itemized)	Not reported; EOR defined radiologically (residual ≤ 0.175 cm^3^) rather than by an explicit intraoperative stopping rule

## Data Availability

No new data were created or analyzed in this study. Data sharing is not applicable to this article.

## Supplementary Materials

The following supporting information can be downloaded at: https://www.mdpi.com/article/10.3390/cancers18203365/s1, Table S1: An overview of search terms used for each database.

## Abbreviations

The following abbreviations are used in this manuscript:

5-ALA	5-Aminolevulinic acid
AC	Awake craniotomy
AF	Arcuate fasciculus
AR	Augmented reality
ASD	Autism spectrum disorder
BCNU	Carmustine
BIS	Bispectral index
CT	Computed tomography
DTI	Diffusion tensor imaging
ECoG	Electrocorticography
EOR	Extent of resection
FAT	Frontal aslant tract
FLAIR	Fluid-attenuated inversion recovery
fMRI	Functional magnetic resonance imaging
GA	General anesthesia
GBM	Glioblastoma multiforme
GTR	Gross total resection
HB	House–Brackmann
HGG	High-grade glioma
IDH	Isocitrate dehydrogenase
iMRI	Intraoperative magnetic resonance imaging
IOM	Intraoperative monitoring
iUS	Intraoperative ultrasound
IV	Intravenous
JBI	Joanna Briggs Institute
KPS	Karnofsky Performance Status
LGG	Low-grade glioma
MEP	Motor-evoked potential
MRI	Magnetic resonance imaging
POD	Postoperative day
PpIX	Protoporphyrin IX
PRISMA	Preferred Reporting Items for Systematic Reviews and Meta-Analyses
SAS	Sleep-awake-sleep
SLF	Superior longitudinal fasciculus
SMA	Supplementary motor area
VR	Virtual reality
WHO	World Health Organization
