# Supplementary material for: 5-Aminolevulinic Acid-Guided Glioma Resection During Awake Craniotomy: Systematic Review of Functional Safety and Surgical Benefit
Source: Cancers (Basel). 2026 Oct 17;18(20):3365. doi: 10.3390/cancers18203365 (PMC13702427; doi:10.3390/cancers18203365)
Supplement: Supplementary file 1 [file cancers-18-03365-s001.zip › cancers-4623673-supplementary.pdf]

Table S1. An Overview of Search Terms Used for Each Database

Database	Search Results	Search Terms
**PubMed**	4713	(("awake"[All Fields] OR "awakeness"[All Fields] OR "awakes"[All Fields] OR "awaking"[All Fields]) AND ("craniotomy"[MeSH Terms] OR "craniotomy"[All Fields] OR "craniotomies"[All Fields])) OR (("awake"[All Fields] OR "awakeness"[All Fields] OR "awakes"[All Fields] OR "awaking"[All Fields]) AND ("brain"[MeSH Terms] OR "brain"[All Fields] OR "brains"[All Fields] OR "brain s"[All Fields]) AND ("surgery"[MeSH Subheading] OR "surgery"[All Fields] OR "surgical procedures, opera-tive"[MeSH Terms] OR ("surgical"[All Fields] AND "procedures"[All Fields] AND "operative"[All Fields]) OR "operative surgical procedures"[All Fields] OR "general surgery"[MeSH Terms] OR ("gen-eral"[All Fields] AND "surgery"[All Fields]) OR "general surgery"[All Fields] OR "surgery s"[All Fields] OR "surgerys"[All Fields] OR "surgeries"[All Fields])) OR (("awake"[All Fields] OR "awakeness"[All Fields] OR "awakes"[All Fields] OR "awaking"[All Fields]) AND ("neurosurgery"[MeSH Terms] OR "neurosurgery"[All Fields] OR "neurosurgeries"[All Fields] OR "neurosurgery s"[All Fields] OR "neuro-surgical procedures"[MeSH Terms] OR ("neurosurgical"[All Fields] AND "procedures"[All Fields]) OR "neurosurgical procedures"[All Fields])) OR (("awake"[All Fields] OR "awakeness"[All Fields] OR "awakes"[All Fields] OR "awaking"[All Fields]) AND ("brain mapping"[MeSH Terms] OR ("brain"[All Fields] AND "mapping"[All Fields]) OR "brain mapping"[All Fields]) AND ("awake"[All Fields] OR "awakeness"[All Fields] OR "awakes"[All Fields] OR "awaking"[All Fields]) AND ("cysts"[MeSH Terms] OR "cysts"[All Fields] OR "cyst"[All Fields] OR "neurofibroma"[MeSH Terms] OR "neurofibroma"[All Fields] OR "neurofibromas"[All Fields] OR "tumor s"[All Fields] OR "tumoral"[All Fields] OR "tumor-ous"[All Fields] OR "tumour"[All Fields] OR "neoplasms"[MeSH Terms] OR "neoplasms"[All Fields] OR "tumor"[All Fields] OR "tumour s"[All Fields] OR "tumoural"[All Fields] OR "tumourous"[All Fields] OR "tumours"[All Fields] OR "tumors"[All Fields]) AND ("resect"[All Fields] OR "resectability"[All Fields] OR "resectable"[All Fields] OR "resectates"[All Fields] OR "resected"[All Fields] OR "resect-ing"[All Fields] OR "resection"[All Fields] OR "resectional"[All Fields] OR "resectioned"[All Fields] OR "resectioning"[All Fields] OR "resections"[All Fields] OR "resective"[All Fields] OR "resects"[All Fields])) OR (("craniotomy"[MeSH Terms] OR "craniotomy"[All Fields] OR "craniotomies"[All Fields]) AND ("awake"[All Fields] OR "awakeness"[All Fields] OR "awakes"[All Fields] OR "awaking"[All Fields]))
**Scopus**	5894	ALL (awake AND craniotomy OR awake AND brain AND surgery OR awake AND neurosurgery OR awake AND brain AND mapping AND awake AND tumour AND resection OR craniotomy AND while AND awake)
**Web of Science**	4301	ALL=(awake craniotomy OR awake brain surgery OR awake neurosurgery OR awake brain mapping OR awake tumour resection OR craniotomy while awake)
